# Benefits of fresh strawberries consumption from Huelva in primary dysmenorrhea: a randomized controlled pilot trial

**DOI:** 10.3389/fnut.2026.1934811

**Published:** 2026-09-21

**Authors:** Cynthia Rocío Márquez-Beltrán, María Teresa Iglesias-López, María Jesús Rojas-Ocaña, Elia Fernández-Martínez

**Affiliations:** 1 Doctoral Program in Health Sciences, Universidad de Huelva, Huelva, Spain; 2 Hospital Universitario Juan Ramón Jiménez, Huelva, Spain; 3 Faculty of Health Sciences, Universidad Francisco de Vitoria, Madrid, Spain; 4 Department of Nursing, Universidad de Huelva, Huelva, Spain

**Keywords:** absenteeism, dysmenorrhea, fragaria, menstrual cycle, pelvic pain, strawberry

## Abstract

**Introduction:**

Primary dysmenorrhea (PD) affects a high percentage of women of reproductive age and is one of the most common causes of school and work absenteeism. It significantly reduces quality of life and impairs daily activities. Strawberry consumption has demonstrated benefits in chronic and inflammatory diseases. Therefore, the aim of this study was to evaluate the effectiveness of consuming fresh strawberries from Huelva on menstrual pain in young female university students with primary dysmenorrhea.

**Methods:**

A randomized clinical trial was conducted including 64 participants with PD, who were randomly assigned to either the control (*n* = 31) or intervention group (*n* = 33). The intervention consisted of the daily intake of 250 g of fresh strawberries (*Fragaria × ananassa*, Fortuna cultivar) for 1 month. The primary outcome was pain intensity assessed using the visual analog scale (VAS). The secondary outcomes included the variables menstrual cycle length, bleeding duration, hours of sleep, absenteeism, perception of health status, State–Trait Anxiety Inventory (STAI) and mental well-being. The data were collected at baseline, at 1 month and at 2 months.

**Results:**

A significant decrease in maximum self-reported menstrual pain was observed at one and 2 months compared with baseline in the intervention group. Regarding menstrual cycle duration, a significant between-group difference was observed between the cycle preceding the study and the second cycle, with a greater shortening in the control group. Perceived health status was also significantly higher in the intervention group at one and 2 months compared with the control group.

**Discussion:**

These findings support the recommendation that healthcare professionals promote the consumption of polyphenol-rich foods, such as strawberries, for the relief of menstrual pain.

**Clinical trial registration:**

ClinicalTrials.gov NCT06250660 on 2024/01/26. This clinical trial was funded by the Asociación Onubense de Productores y Exportadores de Fresa, Freshuelva.

## Introduction

The term primary dysmenorrhea (PD) refers to cyclic chronic abdominal pain associated with menstruation, that may occur before, during, or after menses (1–3). It is not associated with any pelvic pathology (2) and is often accompanied by other symptoms such as nausea, dizziness, vomiting, gastrointestinal disturbances, headache, irritability, or depressive symptoms (1, 3, 4).

This condition affects approximately 70% of women of reproductive age worldwide (5), with a higher incidence among women under 25 years of age (1). In southern Spain, 63.3% of university women are estimated to be affected (6).

PD is one of the leading causes of school and work absenteeism (7, 8) and is associated with a reduced quality of life (9, 10). Affected women experience limitations in daily activities, physical exercise, academic performance, and social and sexual relationships (11). However, this condition in underdiagnosed, as many women do not seek medical care, because it is a normal part of the menstrual cycle (1, 7, 10, 12).

The etiology of PD is not fully understood, although the most widely accepted theory involves excessive prostaglandin production. Prostaglandins induce vasoconstriction of uterine blood vessels, leading to abnormal uterine contractility, ischemia, hypoxia and increased sensitivity of nerve endings (7, 10).

The most common treatments are nonsteroidal anti-inflammatory drugs (NSAIDs) and oral contraceptives (OCs) (7, 10, 13). However, their use is limited by side effects, including gastrointestinal, hepatic, renal and cardiovascular risks associated with NSAIDs, weight gain, irregular bleeding, and thromboembolic risk associated with OCs (7). Additionally, approximately 18% of women with dysmenorrhea exhibit resistance to NSAIDs (14, 15).

Consequently, there is growing interest in non-pharmacological alternatives, such as ginger supplements (16), which have been shown to be effective in reducing menstrual pain, as well as others such as exercise-based interventions (17) or, specifically, yoga (7, 18). In terms of diet, consuming two or more fruits per day has been identified as a protective factor against menstrual pain (19).

The benefits of strawberry consumption found in the literature due to its bioactive compounds are related to chronic inflammatory and cardiovascular diseases, cancer and Alzheimer’s disease (20). Its effects on longevity and lean body mass have also been identified (21). Specifically, in a clinical trial conducted by Schell et al. (22), the researchers concluded that strawberries may have analgesic and anti-inflammatory effects on obese adults with knee osteoarthritis.

Strawberries are rich in bioactive compounds, specifically flavonoids, including anthocyanins, and phenolic acids, which have been associated with antioxidants and anti-inflammatory activities (20, 23). These properties could be particularly relevant to PD, whose pathophysiology is closely related to increased prostaglandin synthesis and inflammatory processes (7, 10, 24, 25).

However, most of these studies conclude that further research is needed to determine the optimal dose and duration of intake to have a significant influence on the levels of biomarkers or pathways related to chronic diseases (22, 26–31).

Nevertheless, no evidence was found in studies evaluating the effects of strawberries on menstrual pain. Furthermore, intervention studies with strawberries found in the scientific literature were carried out using freeze-dried strawberry powder (21, 32, 33), due to the complexity of using fresh strawberries, as they are seasonal, perishable, and associated with a significant cost; however, the use of fresh strawberries has been suggested for studying their potential benefits (20).

Despite the above, in the southern region of Spain, local strawberry cultivation predominates; therefore taking into account the availability of the product, and based on the literature reviewed, a randomized controlled trial with fresh strawberries from Huelva was proposed to evaluate their efficacy on the intensity of menstrual pain in young female university students from the University of Huelva (UHU) with PD.

## Methodology

### Study design

A prospective, parallel-group, pilot randomized controlled trial (RCT) was conducted among young female university students from the University of Huelva with PD and compared with a control group. The trial was registered at ClinicalTrials.gov on January 26, 2024 (NCT06250660). The trial protocol is publicly available in the registry and was not modified after its initiation.

No in silico, *in vitro*, animal, or other preclinical studies were conducted as part of this research. The study consisted exclusively of a randomized controlled pilot trial in human participants.

Participants were recruited in March, and the intervention phase took place during April 2024. Data were collected at baseline, after 1 month and after 2 months, ending the follow-up in June 2024.

This clinical trial was conducted according to the 2010 Consolidated Standards of Reporting Trials (CONSORT) guidelines (34) (Figure 1).

**Figure 1:**
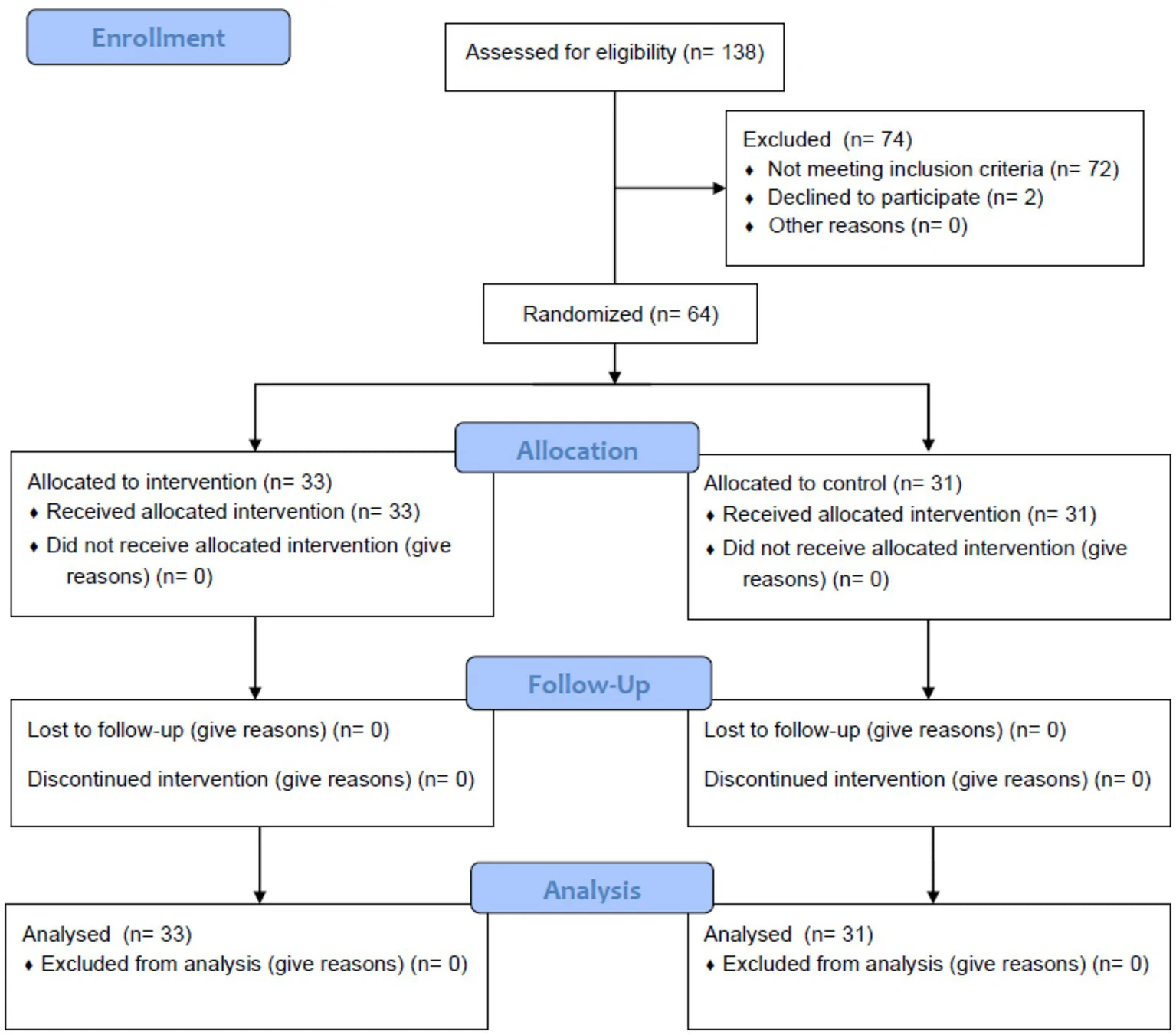
CONSORT 2010 flow diagram.

### Participants

GPower software was used to estimate the minimum sample size, taking into account that the minimum clinically relevant reduction was preset at 1 point on the VAS. With a significance level of 0.05 and a statistical power of 80%, the minimum sample size was estimated at 25 participants per group, which was increased by 20% per group to account for potential dropouts or losses during the study. Therefore, the final pre-specified minimum sample size was 30.

A total of 64 eligible participants were randomized: female students aged from 18 to 35 years old from the University of Huelva who were nulliparous and had PD, which was identified based on self-reported menstrual pain in the absence of a diagnosed gynecological condition, as assessed through the recruitment questionnaire. Participants were also required to report menstrual pain at least once in the last 6 months or three times in the last year (35–37) and not to be using hormonal contraception. In addition, an inclusion criterion was having experienced menstrual pain during the last 3 cycles with a minimum VAS intensity of 4 out of 10, which is considered moderate-to-severe pain according to previous literature on this condition (36, 37). Students were excluded if they were on a mobility program during the study period, had ever had a miscarriage, had any condition or disease that required them to limit the intake of fruits (or strawberries specifically) in their diet, or had suffered from a chronic disease associated with pain. A total of 64 participants were randomized into an intervention group with 33 participants and a control group with 31 participants.

### Randomization and allocation

Participants were assigned 1:1 to the intervention and control groups using a sequence of random numbers generated automatically in Excel (Microsoft Office) without restrictions (no blocking, stratification or minimization). The researchers were unaware of the group assignments at the time of recruitment, which ensured the masking of the assignment until the time of the assignment itself.

Due to the nature of the intervention, blinding was not possible; therefore, an open-label design was used while maintaining randomization to minimize bias and preserve its internal validity.

### Outcome measures

The primary outcome of the study was the effect of strawberry consumption on the intensity of menstrual pain measured using VAS. As complementary outcomes, gynecological aspects (days of menstruation, amount of bleeding and cycle length), absenteeism, impact on daily and academic activities, mental well-being, anxiety state and quality of life were assessed. Data were collected through the recruitment and follow-up form, the VAS (38–40), the Warwick-Edinburgh Mental Wellbeing Scale (WEMWBS) (41, 42), the State–Trait Anxiety Inventory (STAI) (43, 44) and the VAS EuroQol (EQ VAS) (45).

### Interventions

Participants in the control group did not receive any dietary intervention, they continued their usual diet and treatment. In addition to the above, the intervention group consumed a daily supplement of 250 g of fresh strawberries for 1 month. The strawberries belonged to the species *Fragaria* × *ananassa* and the cultivar Fortuna, which is adapted to the agroclimatic conditions of Huelva, Spain (46).

The strawberries were sourced from Huelva, Spain, the same region where the study was conducted, and were supplied directly by local strawberry growers through a Huelva-based association of strawberry growers. The strawberries were delivered to the research team on the same day they were harvested from the field and were subsequently distributed to the participants at the university facilities on the same day. This procedure ensured that the strawberries were provided as a fresh product throughout the intervention.

The research team was responsible for distributing strawberries to the participants, taking into account their preferences regarding location and time when organizing the delivery of the strawberries. Adherence was monitored daily via self-reported diaries. The participants were also followed up via instant messaging and email to support compliance and answer any queries.

### Data collection

Data collection was carried out through self-administered questionnaires in person. Data were collected before the start of the study (baseline data), at 1 month and at 2 months after the beginning of the intervention. The questionnaire was tested for comprehensibility on a group of 10 women beforehand, and the necessary adjustments were made.

### Statistical analysis

Data were analyzed according to protocol group assignment using the statistical package SPSS (Statistical Package for Social Sciences), version 25.0 (47). Quantitative variables are presented as mean, standard deviation or median, and qualitative variables as frequencies and percentages. The Mann–Whitney *U* test was used for non-normally distributed data. *Post hoc* analyses were performed to further explore statistically significant findings. Statistical significance was set at *p* < 0.05.

## Results

### Sample description

The study sample was composed of participants with the youngest aged 19 years and the oldest aged 32 years. No statistically significant differences were found between groups at baseline regarding body mass index (BMI), age at menarche, cycle length, bleeding duration, maximum intensity of menstrual pain measured on the VAS, absenteeism related to menstruation, weekly exercise hours, daily rest hours, or perceived health status on a 0–100 scale, the only baseline difference were found in the mean WEMBS score (Table 1). There were also no differences regarding self-reported menstrual regularity between women in the two groups, with 66.7% of women in the intervention group and 71% in the control group reporting regular periods at the start of the study (*p* = 0.46).

**Table 1:** Baseline characteristics of all participants (mean ± SD).

Variables	Intervention group *N* = 33	Control group *N* = 31	Total (overall sample) *N* = 64	*p*-value^a^
Age (years)	21.67 ± 2.23	21.74 ± 2.54	21.70 ± 2.36	0.978
Weight (kg)	60.76 ± 9.42	63.03 ± 11.20	61.86 ± 10.31	0.463
Height (cm)	163.61 ± 6.52	163.32 ± 6.92	163.47 ± 6.67	0.925
BMI (kg/m^2^)	22.66 ± 1.96	23.70 ± 4.36	23.2 ± 3.70	0.677
Menarche (age in years)	11.94 ± 1.69	12.00 ± 1.24	11.97 ± 1.48	0.912
Length of menstrual cycle (days)	29.88 ± 5.28	31.77 ± 5.80	30.80 ± 5.58	0.686
Duration of menstrual bleeding (days per cycle)	5.24 ± 0.97	5.35 ± 1.36	5.30 ± 1.16	0.894
Intensity of maximum menstrual pain (VAS)	7.61 ± 1.50	7.26 ± 1.13	7.44 ± 1.33	0.235
Absenteeism during last menstruation (days)	0.85 ± 0.67	0.71 ± 0.72	0.8 ± 0.69	0.493
Exercise (weekly hours)	4.00 ± 2.55	4.03 ± 3.92	4.0 ± 3.23	0.628
Rest (daily hours)	6.70 ± 1.07	6.81 ± 1.17	6.75 ± 1.11	0.652
EQ VAS (0–100)	85.69 ± 9.04	79.00 ± 16.90	82.40 ± 13.79	0.066
STAI state	16.83 ± 8.57	18.34 ± 10.35	17.66 ± 9.53	0.694
STAI trait	21.21 ± 9.95	22.90 ± 11.45	22.10 ± 10.70	0.814
WEMWBS	55.22 ± 6.15	51.35 ± 8.75	53.32 ± 7.73	0.048*

a Mann–Whitney *U* test; *p < 0.05.

### Comparison of results in the cycle prior to the intervention, 1 month, and 2 months

#### Intensity of menstrual pain

Baseline menstrual intensity, taking into account the first day of menstruation (which often coincides with the day of maximum intensity), decreased at 1 month by a mean of 1.91 ± 2.67 in the group that consumed strawberries, compared with a mean decrease of 0.35 ± 2.01 in the control group (*p* < 0.01). On the other hand, when comparing the maximum pain of the cycle perceived during the cycle prior to the study, no differences were found between groups; however, differences were observed in the first and second cycles after the start of the trial. In the intervention group, baseline menstrual pain intensity, taking into account as the maximum self-reported pain, decreased at 1 month by a mean of 1.38 ± 2.25 compared with a mean increase of 0.26 ± 1.79 observed in the control group (*U* = 249.00; *Z* = −3.171; *p* ≤ 0.01). The estimated difference in the median change from baseline was 1.00 points (95% CI: 1.00 to 2.00), with an effect size of *r* = 0.41.

If we compare the decrease between the cycle prior to the study and the second cycle after the study, the mean decreased by 1.42 ± 2.59 in the intervention group compared with an increase of 0.42 ± 1.59 in the control group (*U* = 181.50; *Z* = −2.589; *p* = 0.01), with an estimated difference in the median of 1.00 point (95% CI: 0.00 to 3.00) and *r* = 0.37 (see Table 2).

**Table 2:** Comparison of maximum pain intensity (VAS) in the cycle prior to the intervention, in the first and second cycle after intervention.

	Intervention group	Control group	*p*-value
Prior VAS	7.61 ± 1.50	7.26 ± 1.13	0.235
1° cycle VAS	6.22 ± 2.31	7.52 ± 1.68	0.020*
2° cycle VAS	6.27 ± 2.74	7.75 ± 1.17	0.049*

*p < 0.05 according to the Mann–Whitney *U* test for independent samples.

### Secondary outcomes

Variations in cycle length were identified in women in the intervention group throughout the study. In relation to women in the control group, the duration of the menstrual cycle was significantly shortened, with a more pronounced and significant difference between the prior cycle to the study and the second cycle (intervention group = 0.46 ± 6.65 vs. control group = 5.12 ± 8.70; *p* = 0.035) and a less marked difference between the prior cycle and the first month (intervention group = 3.38 ± 8.70 vs. control group = 6.93 ± 10.91; *p* = 0.323). Regarding perceived health status, no statistically significant differences were observed between groups at baseline (*p* = 0.066); however, perceived health status was significantly higher in the intervention group at 1 month (intervention group = 86.34 ± 17.28 vs. control group 79.69 ± 14.42; *p* = 0.006) and at 2 months (intervention group 88.00 ± 11.52 vs. control group 80.00 ± 14.09; *p* = 0.015). However, no statistically significant differences were found for duration of bleeding, hours of sleep, anxiety, mental well-being, STAI state, or absenteeism when comparing the differences between baseline scores and those from the first cycle, or between baseline scores and second cycle scores between the two groups (Table 3).

**Table 3:** Comparison of variables between the pre-trial cycle, at one month and at two months.

		Intervention group	Control group	*p*-value^a^
Cycle length	Basal	29.88 ± 5.28	31.77 ± 5.81	0.686
	1 month	26.50 ± 9.52	24,71 ± 9.21	0.460
	2 months	29.35 ± 8.75	24.96 ± 8.94	0.027*
Duration of bleeding	Basal	5.24 ± 0.97	5.35 ± 1.36	0.894
	1 month	4.88 ± 0.942	5.21 ± 1.18	0.275
	2 months	5.00 ± 0.90	5.16 ± 1.21	0.569
Hours of sleep	Basal	6.70 ± 1.07	6.81 ± 1.17	0.652
	1 month	6.64 ± 0.92	6.81 ± 1.01	0.607
	2 months	6.61 ± 0.82	6.56 ± 1.13	0.893
Absenteeism	Basal	0.85 ± 0.671	0.71 ± 0.717	0.493
	1 month	0.50 ± 0.577	1.00 ± 1.225	0.730
	2 months	0.00 ± 0.00	0.33 ± 0.516	0.476
Perceived health status	Basal	85.69 ± 0.04	79.00 ± 16.9	0.066
	1 month	86.34 ± 17.28	79.69 ± 14.42	0.006*
	2 months	88.00 ± 11.52	80.00 ± 14.09	0.015*
STAI state	Basal	16.83 ± 8,57	18.34 ± 10.35	0.694
	1 month	14.43 ± 8.16	18.42 ± 10.96	0.205
	2 months	17.63 ± 12.32	23.72 ± 14.35	0.133
WEMWBS	Basal	55.22 ± 6.15	51.35 ± 8.75	0.048*
	1 month	54.54 ± 6.15	52.24 ± 8.83	0.175
	2 months	54.85 ± 8.56	51.60 ± 10.16	0.351

a Mann-Whitney *U* test for independent samples; *p < 0.05.

No adverse events related to the intervention were reported during the study period.

## Discussion

This study aimed to evaluate the potential efficacy of a dietary intervention consisting of daily consumption of fresh strawberries (*Fragaria* × *ananassa*, Fortuna cultivar) to alleviate menstrual pain. A decrease in menstrual pain was observed at 1 month and 2 months after the start of the intervention.

In line with this, the literature contains evidence of the efficacy of other studies that demonstrated potential benefits of other polyphenol-rich foods in relieving menstrual pain. Adib Rad et al. (16) reported beneficial effects of an intervention with ginger. On the other hand, Zeraati et al. (48) reported that fennel supplementation may be effective for the relief of PD. Additionally, Jahangirifar et al. (49), demonstrated that cinnamon may reduce the intensity of PD.

Furthermore, since the intervention was carried out with 250 g of fresh strawberries per day, the findings are consistent with the literature suggesting that consuming 2 or more pieces of fresh fruit per day is associated with a decreased intensity of menstrual pain (19). Likewise, our result is in line with the study published by Schell et al. (22), which demonstrated pain relief in a different condition, in this case patients with osteoarthritis, through an intervention based on strawberries. However, the present study differs in that strawberries were consumed fresh, despite the logistical complications involved, as strawberries are a perishable product.

Regarding other variables, it is noteworthy that the duration of the menstrual cycle was shortened among young women who were not subjected to the intervention. This finding could be attributable to the fact that university students are exposed to stress, as extensive literature links stress with a shortened menstrual cycle (50–52). It is possible that strawberry consumption may have contributed to modulating the effect of student stress on the menstrual cycle. However, no differences were found in relation to anxiety or mental well-being. This finding may be explained by the lack of medium- and long-term results, which is when improvements in mental health variables are usually observed (53), with the scientific literature reporting a progressive improvement over time in symptoms of depression and anxiety from 6 months onward (54). There were also no significant changes in relation to perceived health: this may also be attributable to the fact that intervention was limited to 4 weeks, and the follow-up period was 2 months from the start of the period. Despite no significant difference in absenteeism between the intervention and control groups at baseline, 1 month and 2 months, a noticeable reduction in absenteeism rates was observed in the intervention group, reaching a value of zero at 2 months. This result may suggest a potential positive impact of the intervention, and the lack of statistical significance could be related to the sample size. Along these lines, it could be proposed to implement a longer intervention and evaluate the impact in the medium and long term.

The main limitations of the study are the limited sample size—due to it being contextualized in a pilot study-, the intervention time was 4 weeks, and the follow-up was limited to 2 months with self-reports on their last menstruations. The fact that the women knew which group they belonged to, and that the main variable (menstrual pain intensity) was self-reported, represents intrinsic biases very common in intervention studies on menstrual pain. Another limitation worth noting is a baseline difference between groups in the mean WEMBS score. We aim to be able to incorporate other objective variables in future studies, with a larger sample size and including outcome variables such as inflammatory biochemical indicators and microbiota analysis.

As strengths, we highlight that this is a pioneering study with fresh strawberries, which the population describes as having a pleasant taste and, therefore, being easy to include in the daily diet of the population. Furthermore, it is a food whose consumption has already demonstrated metabolic health benefits (23, 55, 56). Previous studies conducted with the Fortuna cultivar used in the present study have reported relevant nutritional and quality characteristics, including ascorbic acid, phenolic compounds and anthocyanins, as well as favorable physicochemical properties (46, 57). These results are encouraging and support further research to contribute to the relief of menstrual pain in women with PD, given the health and socioeconomic impact that this condition involves.

It seems reasonable that, based on previous literature and the findings of this pilot study, healthcare professionals could include polyphenol-rich foods among dietary recommendations as for women with menstrual pain. This could include fresh strawberries, as well as dark chocolate with a high percentage of cocoa (greater than 70%) (58), turmeric (59), or dried figs (60), among others, and the consumption of two or more pieces of fruit per day (19). As most women with PD do not consult healthcare professionals regarding their menstrual pain (61, 62), raising awareness among the general population through other settings, such as the school environment, may also be warranted.

## Conclusion

Daily consumption of fruit, specifically fresh strawberries, may reduce the intensity of pain in PD, making it a suitable non-pharmacological strategy for alleviating menstrual pain in young women affected by this condition.

However, given the inherent limitations of the study design, it is also important to continue along these lines through clinical trials with larger sample sizes and longer follow-up periods. This would help to expand the available scientific evidence on non-pharmacological methods for alleviating this condition, which significantly affects the quality of life of so many women.

## Data Availability

The raw data supporting the conclusions of this article will be made available by the authors, without undue reservation.
